# A Dual-Branch Lightweight Network for Multimodal Image Fusion with Mamba and INN

**DOI:** 10.3390/s26123814

**Published:** 2026-06-15

**Authors:** Nan Li, Hongxin Li, Lin Tian

**Affiliations:** 1 Xinjiang Laboratory of Phase Transitions and Microstructures in Condensed Matter Physics, Yili Normal University, Yining 835000, China; 20240050186@ylnu.edu.cn (N.L.); 20240050188@ylnu.edu.cn (H.L.); 2 School of Electronic Engineering, Yili Normal University, Yining 835000, China

**Keywords:** multimodal image fusion, lightweight network, Mamba, invertible neural network, infrared-visible image fusion, medical image fusion

## Abstract

Multimodal image fusion aims to integrate complementary information from heterogeneous imaging modalities into a single informative image. However, many deep learning-based fusion methods rely on complex feature extractors, leading to high computational cost and limited suitability for real-time deployment on resource-constrained devices. To address this issue, this paper proposes a lightweight Mamba-INN dual-branch network for efficient multimodal image fusion. The proposed model decouples global structure modeling from local detail preservation. A simplified Mamba-inspired branch is designed to capture long-range contextual dependencies, while a lightweight invertible neural network branch preserves high-frequency textures and edge information through information-preserving transformations. The lightweight INN branch preserves high-frequency texture and edge information during the forward feature transformation process through reversible feature partitioning, coupled transformations, and exponential scale modulation, thereby reducing the loss of detail caused by feature compression. Compact shallow feature refinement, module reuse, low-dimensional channel design, and a streamlined decoder are further introduced to reduce redundant computation. Experiments on infrared-visible and medical image fusion benchmarks, including MSRS, TNO, RoadScene, MRI-CT, MRI-PET, and MRI-SPECT datasets, demonstrate that the proposed method achieves competitive fusion quality with low model complexity. The proposed method achieves performance comparable to or better than that of methods such as CDDFuse, U2Fusion, CNN and SDNet on metrics including MI, VIF, Qabf, and SSIM for infrared-visible and medical image fusion tasks, while containing only 0.24 million parameters and requiring 24.04 GFLOPs of computational power at an input resolution of 256 × 256. Compared to CDDFuse, our method significantly reduces model complexity, enhancing the potential for lightweight deployment while maintaining fusion quality.

## 1. Introduction

Multimodal image fusion aims to integrate complementary information from images acquired by different sensors or imaging mechanisms into a single informative representation. By combining modality-specific advantages, the fused image can provide richer semantic information, clearer structural details, and improved visual perception. Owing to these advantages, multimodal image fusion has been widely applied in infrared-visible image fusion, medical image fusion, remote sensing image fusion, and perception tasks in unmanned systems [1,2,3,4]. In particular, infrared-visible image fusion can simultaneously preserve salient thermal targets from infrared images and texture-rich background details from visible images, thereby improving target recognition and scene understanding in complex environments [1,5,6]. Similarly, medical image fusion integrates complementary structural and functional information from different imaging modalities, such as MRI, CT, PET, and SPECT, providing a more comprehensive visual basis for clinical diagnosis [7,8].

In recent years, deep learning has significantly promoted the development of multimodal image fusion. Representative methods include the autoencoder-based DenseFuse [2], the decomposition-based DIDFuse [3], the unified unsupervised fusion framework U2Fusion [4], the real-time squeeze-and-decomposition network SDNet [9], the detection-oriented TarDAL [5], and the correlation-driven dual-branch feature decomposition network CDDFuse [1]. These methods have achieved promising performance in information preservation, target enhancement, and texture restoration. Nevertheless, as network architectures become increasingly complex, many existing methods still suffer from excessive model parameters and high computational costs. Moreover, insufficient coordination between global contextual modeling and local detail extraction may lead to structural inconsistency, texture loss, or limited fusion quality. These limitations restrict the deployment of existing fusion models on embedded devices, mobile terminals, and real-time perception systems.

Although existing deep fusion methods achieve satisfactory results in object enhancement, texture preservation, and structural restoration, their practical application remains limited by two key issues. First, many methods rely on deep CNNs, Transformers, or complex feature decomposition modules, resulting in high parameter counts, FLOPs, and inference latency, which makes them difficult to deploy on embedded platforms and mobile devices. Second, some methods fail to adequately balance global structural modeling with local detail preservation, often resulting in issues such as insufficient object salience, blurred edge textures, or poor structural consistency. Therefore, the central research question addressed in this paper is how to simultaneously achieve global semantic modeling and local high-frequency information preservation with low computational overhead.

Recently, FusionMamba [10] introduced Mamba into multimodal image fusion via a dynamic feature enhancement mechanism, demonstrating the effectiveness of state-space models in fusion tasks; Spatial-Frequency Enhanced Mamba [11] further enhanced multimodal feature representations in both the spatial and frequency domains, improving fusion quality; DLiteNet [12], meanwhile, demonstrated the potential of compact networks in cross-modal remote sensing tasks through a dual-branch lightweight architecture. However, the aforementioned methods either focus on Mamba feature enhancement or are primarily tailored to specific remote sensing scenarios, and have not yet fully addressed the challenge of achieving a balanced trade-off between global structural modeling, local detail preservation, and model complexity in multi-type fusion tasks.

Transformers have been widely introduced into low-level vision and image fusion tasks due to their strong capability in modeling long-range dependencies [13,14,15]. For instance, Restormer achieves excellent performance in image restoration by efficiently modeling channel-wise self-attention [15], while Swin Transformer and SwinFusion demonstrate the effectiveness of window-based attention mechanisms in visual representation and image fusion tasks [13,16]. However, the computational complexity of self-attention generally increases substantially with image resolution, which poses challenges for high-resolution image fusion and lightweight deployment. Recently, state-space models, especially Mamba, have attracted increasing attention because of their ability to model long-range dependencies with near-linear computational complexity through selective state updates [17,18,19]. Therefore, introducing Mamba-inspired modeling into image fusion is a promising direction for enhancing global structural representation while maintaining computational efficiency.

In addition to global modeling, local detail preservation is also essential for high-quality multimodal image fusion. Invertible neural networks (INNs), which are characterized by reversible mappings and information-preserving properties, have been applied to image restoration, image rescaling, image hiding, and multimodal image fusion [20,21,22,23]. In CDDFuse, INNs are employed to extract high-frequency detail features, effectively alleviating detail degradation during feature transformation and reconstruction [1]. However, INNs are generally more suitable for modeling local textures and edge information, while their ability to capture global structures and long-range dependencies remains limited. Therefore, a collaborative design that combines the global modeling capability of Mamba-inspired modules with the detail-preserving ability of INNs provides a feasible solution for lightweight and high-quality multimodal image fusion.

The fundamental premise of this paper is that by assigning the modeling of long-range dependencies to the lightweight Mamba branch and the preservation of high-frequency details to the reversible INN branch, while reducing redundant parameters through module reuse, the model can maintain or enhance the information retention, structural consistency, and texture clarity of the fused images while significantly reducing complexity.

Unlike CDDFuse, which employs Lite Transformer for basic feature modeling, this paper uses a simplified Mamba module to replace the attention-based global modeling unit, thereby capturing long-range contextual dependencies with lower computational complexity. Meanwhile, the INN branch leverages the properties of reversible mappings to preserve local texture, edges, and fine-grained structural information, thereby forming a complementary dual-branch mechanism where “Mamba handles global structure and INN handles local details.”

Motivated by the above observations, this paper proposes a lightweight Mamba-INN dual-branch network for multimodal image fusion, building upon the dual-branch feature decomposition paradigm of CDDFuse. Unlike CDDFuse, which adopts a Lite Transformer to extract base features and an INN to extract detail features [1], the proposed method introduces a simplified Mamba-inspired branch for global base feature modeling and a lightweight INN branch for local detail extraction. Through this decoupled design, the proposed network effectively coordinates global structural representation and high-frequency detail preservation. Furthermore, module reuse, low-dimensional channel design, and a compact decoding structure are incorporated to further reduce model parameters and computational complexity while maintaining competitive fusion quality.

Our main contributions are fourfold:

1. To address the high computational complexity of existing methods, we propose a lightweight Mamba-INN dual-branch architecture.
2. To address the lack of coordination between global and local information, we design a Mamba global branch and an INN detail branch.
3. To address parameter redundancy during the fusion stage, we introduce module reuse and a compact decoder.
4. We validate performance and efficiency through multi-dataset, complexity, ablation, and cross-task testing.

## 2. Related Work

Existing fusion methods generally involve a trade-off between fusion quality and computational efficiency: complex models typically achieve better representation of texture and structure, but come with higher parameter counts, greater GPU memory usage, and longer inference latency; lightweight models, while easier to deploy, may sacrifice detail preservation and global consistency. Therefore, constructing a fusion network that combines global modeling capabilities, local detail preservation, and low computational complexity remains a key challenge.

### 2.1. Deep Learning-Based Multimodal Image Fusion

Due to their strong ability to represent nonlinear features, deep learning methods have become a major research focus in multimodal image fusion. Early deep fusion methods typically employed an encoder-fusion-decoder architecture, in which source images are first mapped to a latent feature space, then integrated using specific fusion strategies, and finally reconstructed into a fused image. DenseFuse [2] employs an autoencoder architecture to extract features from infrared and visible-light images, demonstrating the effectiveness of deep feature representations in fusion tasks. While this method features a relatively simple structure and a stable training process, its feature extraction capabilities are limited, and it still struggles to preserve fine-grained textures and salient objects in complex scenes.

DIDFuse [3] employs an image decomposition approach to partition the source image into different frequency components, thereby enhancing the interpretability of the fusion process and facilitating the separate processing of structural and detail information. However, decomposition strategies typically introduce additional computational steps, and their performance is susceptible to variations in decomposition quality. U2Fusion [4] proposes a unified unsupervised fusion framework that is applicable to various image fusion tasks and demonstrates good task generalization capabilities. However, because its design prioritizes generality, it does not sufficiently exploit specific complementary relationships between different modalities, which may limit its performance in preserving complex structures or high-frequency details.

SDNet [9] improves fusion efficiency through structural compression and decomposition, embodying a lightweight design philosophy tailored for real-time applications. Its advantages lie in fast inference speeds and strong deployment potential; however, due to the compression of the network’s expressive power, it may fall short in preserving textural details and reconstructing complex structures. TarDAL [5] incorporates object detection information into the fusion process, making the fusion results more beneficial for downstream perception tasks and enhancing the saliency of infrared objects. However, such task-driven methods typically rely on additional task constraints or supervisory signals, entail high training complexity, and may face limitations when generalized to other fusion scenarios. RFNet and ReCoNet further consider the relationship between registration and fusion, improving robustness in complex scenes or under non-strict registration conditions; however, joint modeling also increases the complexity of the network architecture and computational workflow [6,24].

In recent years, dual-branch and feature decomposition-based methods have garnered attention. CDDFuse [1] effectively enhances the retention of complementary multimodal information by modeling fundamental features and detailed features separately through a correlation-driven dual-branch feature decomposition network. The advantage of this method lies in its ability to effectively distinguish between global structural information and local textural information, providing valuable design insights for subsequent dual-branch fusion networks. However, CDDFuse still relies on relatively complex feature modeling modules, and the number of parameters and computational load remain significant, posing challenges for deployment on resource-constrained devices.

Overall, deep learning-based fusion methods have significantly improved the quality of fused images, but performance gains often depend on more complex network architectures. While complex models offer stronger feature representation capabilities, they impose a heavy computational burden; lightweight models, though more efficient, may sacrifice detail preservation and structural consistency. Therefore, designing a fusion network that maintains sufficient feature representation while reducing computational complexity is a prerequisite for practical deployment.

### 2.2. Transformer and Mamba-Based Global Modeling

Long-range dependency modeling is essential for image fusion because multimodal images often contain complementary information distributed across different spatial regions. Transformers, which rely on self-attention mechanisms, have shown strong capability in capturing global contextual relationships and have been widely used in image restoration, recognition, and fusion tasks [13,16]. Restormer designs an efficient Transformer architecture for high-resolution image restoration and improves the balance between local and global feature modeling through channel-wise self-attention [15]. Swin Transformer reduces the computational burden of standard self-attention by using shifted window attention, and SwinFusion further demonstrates the effectiveness of window-based attention in general image fusion tasks [13,16]. These studies indicate that attention-based models can effectively enhance structural consistency and contextual representation in fused images.

However, the computational cost of attention mechanisms remains a major limitation, especially when dealing with high-resolution images. Even with window partitioning or channel-wise attention, Transformer-based methods may still introduce considerable memory consumption and computational overhead. This limits their practicality in real-time image fusion and lightweight deployment scenarios. Recently, state-space models have provided a new perspective for efficient sequence modeling. Mamba, based on selective state-space modeling, can capture long-range dependencies with near-linear computational complexity [17]. Subsequent studies, such as Vision Mamba and VMamba, have extended state-space modeling to visual tasks and verified its potential for efficient visual representation learning [18,19]. Compared with conventional self-attention, Mamba-style modeling offers a more lightweight alternative for global feature extraction. This makes it particularly attractive for multimodal image fusion, where both global structural consistency and computational efficiency are required.

In summary, both Transformer and Mamba can enhance global structural modeling capabilities, but they have different limitations in terms of computational efficiency and detail preservation. While the Transformer excels at global modeling, it incurs significant computational overhead; conversely, Mamba offers higher modeling efficiency but lacks the ability to preserve local details. Therefore, combining Mamba’s efficient global modeling capabilities with a local detail preservation module represents a viable approach to achieving high-quality, lightweight fusion.

### 2.3. Invertible Neural Networks for Local Detail Preservation

In addition to global structural modeling, preserving local details such as edges, textures, and fine anatomical boundaries is equally important for multimodal image fusion. Invertible neural networks originated from normalizing flow-based models and are characterized by reversible transformations between inputs and outputs [20,21]. Because of their information-preserving nature, INNs have been widely explored in image rescaling, image hiding, super-resolution, restoration, and image fusion tasks [22,23,25]. Unlike conventional feed-forward networks, INNs can reduce information loss during feature transformation, which is particularly beneficial for fusion tasks that require the simultaneous preservation of source-modality information.

In the field of multimodal image fusion, CDDFuse introduces INNs to extract high-frequency detail features, thereby alleviating texture degradation and edge loss during the fusion process [1]. This demonstrates that reversible structures are effective in maintaining local information fidelity. Nevertheless, INNs are mainly advantageous in modeling local textures and detailed spatial variations, while their ability to represent global contextual relationships is relatively limited. For fusion scenarios involving complex scenes, large-scale structures, or long-range dependencies, relying solely on INN-based feature extraction may be insufficient. Therefore, combining INNs with a global modeling module provides a more balanced solution: the global branch captures structural dependencies, while the INN branch preserves fine-grained details.

Based on the above analysis, INNs are better suited to serve as modules for preserving local details, working in tandem with branches that possess global modeling capabilities. The global branch is responsible for capturing long-range dependencies and overall structural information, while the INN branch is used to preserve texture, edges, and high-frequency details. The complementary nature of these two approaches allows for a better balance between structural consistency and detail fidelity.

### 2.4. Lightweight Design for Efficient Image Fusion

Lightweight design is crucial for the practical deployment of multimodal image fusion models. In real-world applications such as unmanned systems, edge perception, mobile medical diagnosis, and remote sensing platforms, fusion algorithms are often expected to operate under limited computational resources and strict latency constraints. Existing lightweight methods usually reduce model complexity by decreasing network depth, reducing channel dimensions, adopting depthwise separable convolutions, simplifying fusion modules, or designing compact encoder–decoder structures [9,26]. These strategies can effectively reduce the number of parameters and FLOPs, making image fusion models more suitable for resource-constrained environments.

However, excessive compression may weaken the feature representation capability of the network. In image fusion tasks, insufficient representation often leads to problems such as blurred edges, weakened target saliency, loss of texture details, or structural distortion. With this in mind, combining the simplified Mamba module with the lightweight INN module is a promising approach. The Mamba branch can capture global contextual dependencies with lower computational complexity, while the INN branch can preserve local high-frequency texture and edge information through reversible transformations. At the same time, through module reuse, low-dimensional channel design, and compact decoders, redundant computations and parameter scales can be further reduced, thereby achieving a better balance between fusion quality and computational efficiency.

In summary, existing multimodal image fusion methods primarily face the following three challenges. First, many deep learning methods rely on complex encoders, feature decomposition modules, or attention mechanisms to improve fusion quality, but this often results in high parameter counts and computational overhead, limiting their deployment in resource-constrained scenarios. Second, global modeling methods such as Transformers and Mamba can effectively capture long-range dependencies, but Transformers have high computational complexity, while using Mamba alone may struggle to fully preserve local high-frequency details. Third, INN-based methods possess good information retention capabilities and are suitable for modeling local textures and edge details, but their ability to express global structures is relatively limited.

Therefore, high-quality and efficient multimodal image fusion requires a complementary modeling framework rather than a single feature extraction mechanism. Specifically, a fusion network should possess both efficient global structural modeling capabilities and local detail preservation capabilities, while minimizing redundant parameters and unnecessary computational overhead. Motivated by this, we propose a lightweight Mamba-INN dual-branch multimodal image fusion network. Specifically, the Mamba branch is used to efficiently capture global structure and long-range contextual dependencies, while the INN branch preserves local texture, edges, and high-frequency details through reversible transformations. Additionally, this paper further introduces module reuse, low-dimensional channel design, and a compact decoder to reduce model complexity. Through these designs, the proposed method aims to achieve a better balance among fusion quality, structural consistency, detail preservation, and computational efficiency.

## 3. Methodology

This section presents the proposed lightweight dual-branch feature fusion framework for multimodal image fusion. The method is designed to address the limitations of existing fusion networks, including excessive parameter scale, high computational complexity, and limited deployability on resource-constrained devices. Following the principle of lightweight modeling, the proposed framework combines the efficient global modeling capability of a simplified Mamba-inspired module with the detail-preserving property of a lightweight invertible neural network (INN). In this way, the network can achieve complementary feature representation while maintaining low computational overhead.

### 3.1. Overview

**Figure 1:**
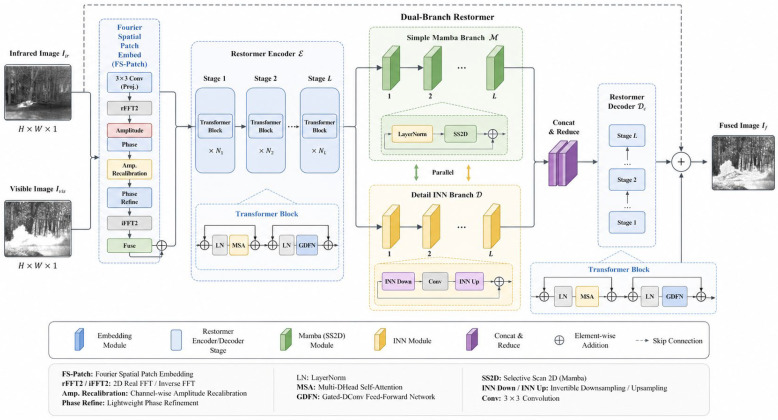
Model framework diagram.

The overall architecture of the proposed lightweight decoupled image fusion network is illustrated in Figure 1. The network follows the general encoder–fusion–decoder paradigm, while its internal components are carefully redesigned to improve computational efficiency and enhance the complementarity between global and local features.

The FS-Patch embedding shown in Figure 1 refers to the Fast Shallow-Space Patch Embedding module, which maps a single-channel source image to a compact feature space without altering the resolution of the input space. This module enhances local continuity between adjacent pixels through 3 × 3 overlapping convolutions, providing shared shallow-level features for the subsequent Mamba and INN branches, thereby avoiding the additional computational overhead associated with redundant encoding in the dual-branch architecture.

Given a pair of registered source images $I_{1}$ and $I_{2}$, the network first obtains shallow feature representations through a lightweight shared encoding module. The extracted shallow features are then fed into two parallel branches. Specifically, the simplified Mamba branch is responsible for modeling low-frequency global base features, while the lightweight INN branch focuses on extracting high-frequency local detail features. After that, the base and detail features are fused by the corresponding fusion modules. Finally, the decoder reconstructs the fused representation into the output image.

### 3.2. Encoder

The encoder serves as the primary module for feature extraction, and its design directly affects both the representation quality and the lightweight property of the entire network. Instead of using a deep multi-stage encoder with heavy feature transformations, this paper adopts a compact encoder consisting of overlapping patch embedding, shallow feature refinement, and dual-branch lightweight feature extraction. By introducing module simplification, feature sharing, and compact channel design, the encoder reduces redundant computation while retaining sufficient feature representation ability.

#### 3.2.1. Lightweight Patch Embedding Module

To project the input image into a high-dimensional feature space with low computational cost, a lightweight overlapping patch embedding module is employed. Unlike conventional embedding modules that rely on multiple convolutional layers, this module uses a single convolutional layer to perform feature embedding, thereby reducing parameter redundancy at the initial feature extraction stage. The mathematical expression for the lightweight overlapping block embedding module is:

(1) $$F_{embed}=P(I)=W_{p}\times I$$

where P(⋅) denotes the block embedding operation; $W_{p}\in {\mathbb{R}}^{C_{out}\times C_{in}\times 3\times 3}$ represents the weights of the embedding module’s convolutional kernels; $C_{in}$ denotes the number of channels in the input image; $C_{out}$ denotes the dimension of the embedded feature channels; $F_{embed}\in {\mathbb{R}}^{B\times C_{out}\times H\times W}$ denotes the embedded feature map.

**Figure 2:**
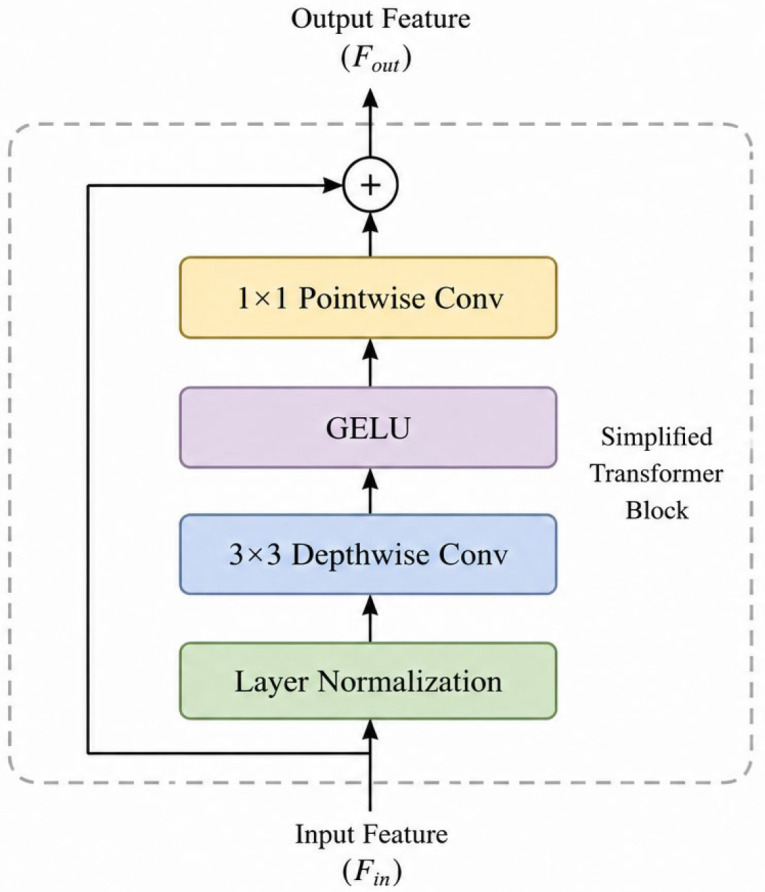
Simplified Transformer block (shallow feature extraction).

As shown in Figure 2, the proposed shallow feature extraction block differs from conventional Transformer structures. Instead of using computationally expensive self-attention, it adopts a lightweight combination of layer normalization, 3 × 3 depthwise convolution, and 1 × 1 pointwise convolution. This structure decouples spatial feature extraction and channel interaction, allowing the network to refine shallow features with limited parameter overhead. Residual connections are further introduced to stabilize feature propagation and maintain representation robustness.

This module employs a single-layer 3 × 3 convolutional kernel for feature embedding. By setting the stride to 1 and padding to 1, it ensures the output feature map dimensions match the input image, thereby avoiding computational overhead from subsequent interpolation operations. Additionally, an unbiased design is adopted to further reduce the number of parameters. Its mathematical expression is:

(2) $$X_{embed}=C_{3\times 3}(X_{in};s=1,p=1,b=False)$$

where $C_{k\times k}(\cdot ;s,p,b)$ denotes the k×k convolution operation, s is the stride, p is the padding and b indicates whether a bias term is included.

#### 3.2.2. Shallow Feature Extraction Module

After patch embedding, a shallow feature extraction module is introduced to refine the embedded features. This module consists of four stacked simplified Transformer blocks. Each block follows a compact structure composed of layer normalization, depthwise convolution, pointwise convolution, nonlinear activation, and residual connection. Compared with standard Transformer blocks, this design removes the self-attention operation and therefore substantially reduces computational complexity.

For a single simplified Transformer block, the core forward propagation logic is as follows: First, the input features are normalized using LayerNorm [15,26] to even out the feature distribution; next, a 3 × 3 depthwise convolution is used to extract local spatial features, which reduces the number of parameters by a factor of $1/C_{\mathrm{out}}$ compared to standard convolution; then, the GELU activation function is introduced to add nonlinearity to the features [27]; next, a 1 × 1 pointwise convolution is applied to adjust the feature channel dimensions, thereby enabling cross-channel feature interaction; finally, a residual connection is applied to the input features to prevent the vanishing gradient problem in deep networks and ensure effective propagation of deep-layer features. The mathematical expression for the simplified Transformer block is:

(3) $$F_{refine}=T(F_{embed})=F_{embed}+P_{1\times 1}\left(G\left(D_{3\times 3}\left(N(F_{embed})\right)\right)\right)$$

where T(⋅) denotes a simplified Transformer block operation, N(⋅) denotes the normalization operation in an adaptive layer, $D_{3\times 3}(\cdot )$ denotes a 3 × 3 depthwise convolution operation, G(⋅) denotes the GELU activation function, $P_{1\times 1}(\cdot )$ denotes a 1 × 1 pointwise convolution operation, and $F_{refine}$ denotes the enhanced shallow-layer refined features.

The final term in Equation (3), which is added to the input features, corresponds to the residual connection shown in Figure 2. This residual path allows shallow embedding features to be directly passed to the output, thereby mitigating the issues of gradient vanishing and detail loss that may arise from stacking multiple layers of lightweight blocks.

The shallow feature extraction module consists of four stacked simplified Transformer blocks, and its overall mathematical expression is:

(4) $$F_{shallow}=T_{4}{}^{\circ}T_{3}{}^{\circ}T_{2}{}^{\circ}T_{1}(F_{embed})$$

where $T_{i}(\cdot )$ denotes the *i*-th simplified Transformer block, and $F_{shallow}\in {\mathbb{R}}^{B\times C_{out}\times H\times W}$ represents the final shallow refined features, which serve as the shared input for the subsequent dual-branch feature extraction module.

#### 3.2.3. Dual-Branch Lightweight Feature Extraction

To achieve the separation and extraction of fundamental and fine-grained features, a dual-branch lightweight feature extraction architecture is designed. It comprises a fundamental feature branch based on a simplified Mamba [17,19] block and a fine-grained feature branch based on a streamlined reversible module. Both branches share shallow refined features $F_{shallow}$, thereby avoiding computational overhead caused by redundant feature extraction.

**Figure 3:**
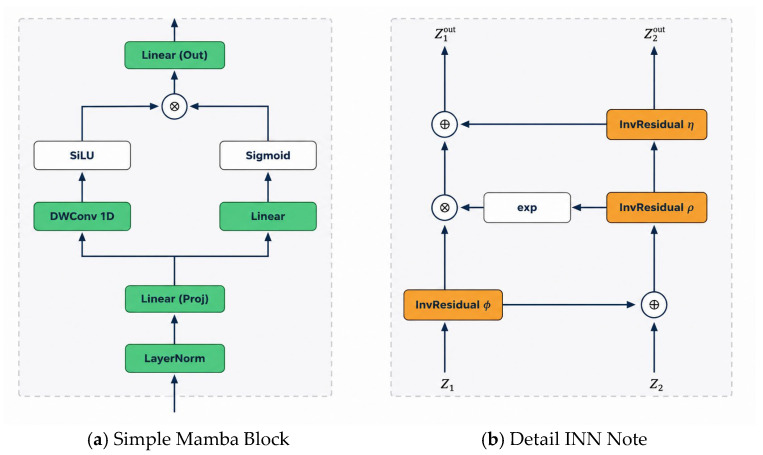
Schematic diagram of the core unit structure in the dual-branch feature extraction module.

As illustrated in Figure 3, the simplified Mamba block and the detail INN node form the two core units of the dual-branch module. The simplified Mamba block adopts a lightweight gated structure based on layer normalization and linear projection. A one-dimensional depthwise convolution and a sigmoid gating mechanism are incorporated to model global contextual relationships and long-range dependencies with limited parameter cost. SiLU is adopted in the Mamba branch because its smooth gating characteristics are better suited for combination with sigmoid gating and one-dimensional deep convolutions to selectively modulate long-range contextual information; meanwhile, GELU is used in the shallow Transformer blocks primarily to enhance the nonlinear representation of local convolutional features. The exponential scaling term in the INN node is used to construct a reversible affine coupling transformation, enabling one set of features to adaptively scale another set. This enhances local texture responses while maintaining the reversibility of the transformation. In contrast, the detail INN node is constructed using reversible residual transformations. Through feature splitting, interaction, and adaptive modulation, the INN branch enhances local detail responses and preserves high-frequency information such as textures, edges, and fine structures. The simplified Mamba branch can be formulated as:

(5) $$F_{mamba}=M(F_{shallow})=F_{shallow}+W_{out}\cdot \left(g_{1}⊙S\left(D_{1D}\left(N(F_{shallow})\right)\right)+(1-g_{1})⊙g_{2}\right)$$

where M(⋅) represents a simplified Mamba block operation, $W_{out}\in {\mathbb{R}}^{C\times C}$ represents linear projection weights, ⋅ represents matrix multiplication, ⊙ represents element-wise multiplication, $g_{1},g_{2}\in {\mathbb{R}}^{B\times L\times C}$ represents the gated vector generated by the linear layer, $D_{1D}(\cdot )$ represents a one-dimensional depth-separable convolution operation, S(⋅) represents the SiLU activation function, and represents the features processed by the Mamba block.

The detail branch is designed to capture high-frequency local information. It is built upon a lightweight INN architecture and consists of three stacked detail node modules. Owing to the reversible mapping property of INNs, the branch can preserve feature information during transformation and reduce the risk of detail loss. This property is particularly suitable for lightweight fusion networks, where excessive feature compression may otherwise weaken texture and edge representation [25]. Because INNs feature bidirectional reversible mappings and computable Jacobians, their feature transformation process can theoretically reduce the information loss caused by irreversible compression. Therefore, using INNs for the detail branch in image fusion helps preserve edge, texture, and local structural information.

The overall forward propagation process in the detail feature extraction branch is as follows: First, the refined shallow-layer feature $F_{shallow}$ is split along the channel dimension into two sub-features, $z_{1}$ and $z_{2}$; then, these two sub-features are fed into a stacked detail node module for reversible interactive enhancement; finally, the enhanced sub-features are concatenated to obtain the final local detail feature $F_{detail}$. The initial feature splitting operation is defined as:

(6) $$z_{1},z_{2}=\mathrm{Split}(F_{shallow}),\;z_{1}\in {\mathbb{R}}^{B\times C/2\times H\times W},\;z_{2}\in {\mathbb{R}}^{B\times C/2\times H\times W}$$

For a single detail node module, the core reversible transformation process is as follows: first, the two sub-features $z_{1}$ and $z_{2}$ are concatenated and then blended via a 1 × 1 convolution to enhance the correlation between sub-features; next, the blended feature is re-segmented to obtain new sub-features ${\hat{z}}_{1}$ and ${\hat{z}}_{2}$; then, a nonlinear transformation based on an inverted residual block is applied to achieve interactive enhancement between the two sub-features; Finally, the enhanced sub-features $z_{1}^{\prime }$ and $z_{2}^{\prime }$ are output. The mathematical expression is:

(7) $$\left\{\begin{array}{l} {\hat{z}}_{1},{\hat{z}}_{2}=\mathrm{Split}\left(C_{1\times 1}\left(\mathrm{Concat}(z_{1},z_{2})\right)\right)\\ z_{2}^{\prime }={\hat{z}}_{2}+\Theta_{\phi }({\hat{z}}_{1})\\ z_{1}^{\prime }={\hat{z}}_{1}⊙\exp\left(\Theta_{\rho }(z_{2}^{\prime })\right)+\Theta_{\eta }(z_{2}^{\prime }) \end{array}\right.$$

where Concat(⋅) is a feature concatenation operation along the channel dimension, $C_{1\times 1}(\cdot )$ is a 1 × 1 convolutional blending operation, $\Theta_{\phi }(\cdot )$, $\Theta_{\rho }(\cdot )$, $\Theta_{\eta }(\cdot )$ are nonlinear transformation operations based on simplified backpropagation blocks, and exp(⋅) is an exponential operation used to perform adaptive scaling of features.

### 3.3. Fusion Layer

The core objective of the feature fusion layer is to achieve effective complementarity and deep integration between global base features $F_{base}$ and local detail features $F_{detail}$, while avoiding the parameter redundancy and computational overhead associated with traditional fusion methods. To this end, the proposed method designs two corresponding fusion components: a base feature fusion layer and a detail feature fusion layer. These two components process global and local features separately, allowing the network to preserve their respective characteristics during fusion.

The basic feature fusion layer takes the global basic features $F_{base}$ as input and employs a simplified Mamba block as its core fusion unit, directly reusing the modular architecture of the encoder’s basic feature branch without the need for additional module design. Leveraging Mamba’s selective sequence modeling capabilities, this layer further refines the global basic features, enhances their global contextual relevance, and achieves adaptive fusion of global structural information.

The detail feature fusion layer takes local detail features $F_{detail}$ as input, employs a simplified INN as its core fusion unit, and directly reuses the modular structure of the encoder’s detail feature branch. Through the reversible transformations of the INN, this layer achieves precise fusion of local detail features, preserving the image’s complete local texture and edge information while preventing the loss of detail caused by over-fusion.

**Figure 4:**
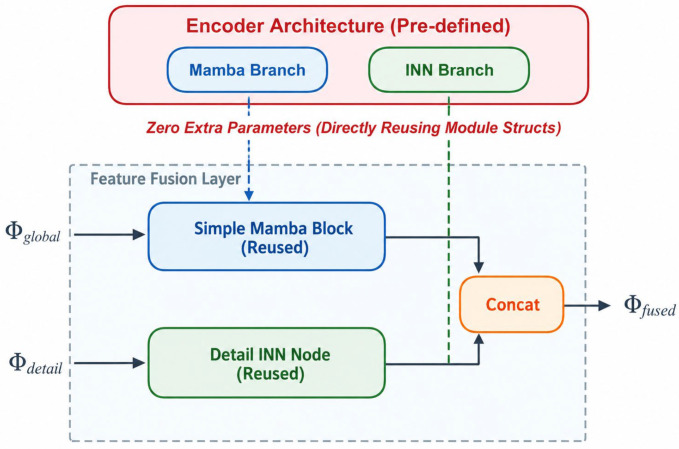
Module Reuse Strategy in Fusion Layer (Parameter Control).

To further reduce the model scale, a module reuse strategy is introduced into the fusion stage, as shown in Figure 4. Instead of designing additional parameter-heavy fusion blocks, the proposed fusion layer reuses the Mamba-inspired and INN-based structures already employed in the encoder. This strategy effectively controls the parameter count and contributes to the extremely compact model size of 0.24 million parameters. More importantly, the reuse strategy allows global context modeling and local detail enhancement to remain consistent between feature extraction and feature fusion.

**Figure 5:**
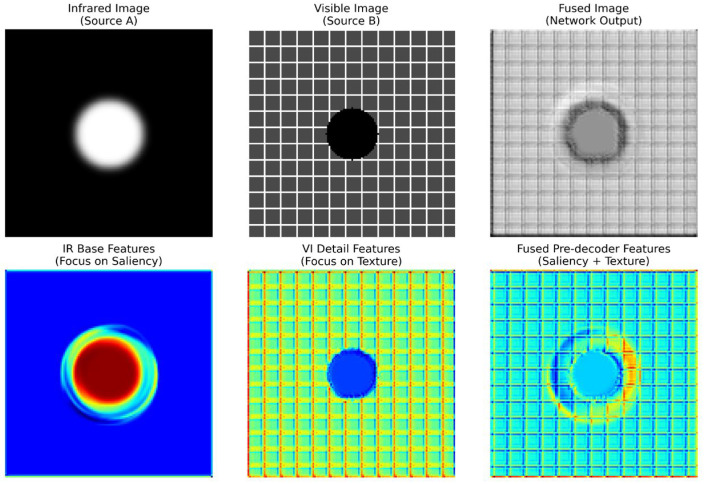
Visualization of the complementary mechanism of dual-branch features.

To analyze the response characteristics of the proposed dual-branch architecture, this paper visualizes the infrared base features, visible detail features, and fused pre-decoded features, as shown in Figure 5. Although these feature maps are generated from an untrained network, they still reveal a preliminary division of labor between the two branches. The base features extracted from the infrared branch exhibit stronger responses in salient target regions, while the activation in large background areas remains relatively weak. This suggests that the base branch tends to emphasize infrared targets and low-frequency object information. In contrast, the detail features extracted from the visible branch show stronger responses around textures, edges, and structured background regions, indicating that the detail branch is more sensitive to high-frequency visual information.

The fused pre-decoded features preserve both target-related responses and texture-related responses. This observation indicates that the proposed dual-branch framework can establish complementary representations of infrared saliency and visible texture information in the feature space. Even before sufficient training, the network already shows a certain tendency toward target enhancement and detail preservation, which supports the structural rationality of combining a Mamba-inspired global branch with an INN-based local branch.

**Figure 6:**
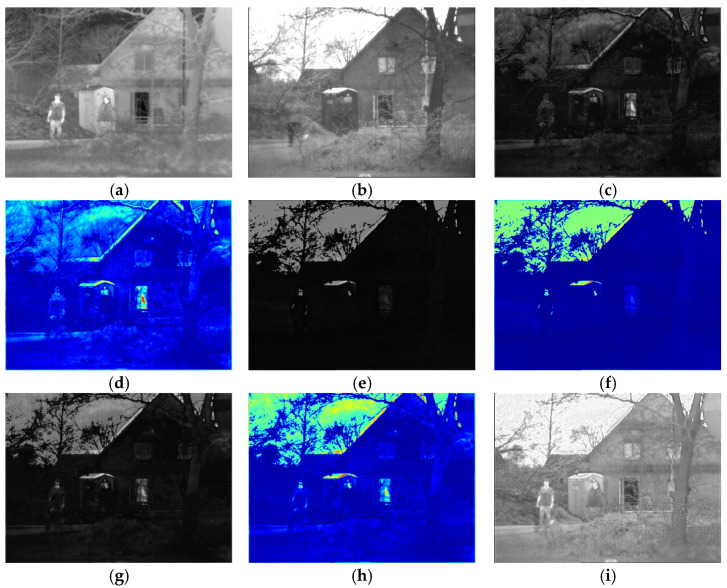
Visualization of branch-wise feature responses generated by the trained model. (**a**) Infrared source image; (**b**) Visible source image; (**c**) Grayscale feature response map of the Mamba branch; (**d**) Heatmap visualization of the Mamba branch feature responses; (**e**) Grayscale feature response map of the INN branch; (**f**) Heatmap visualization of the INN branch feature responses; (**g**) Grayscale feature response map of the fused features; (**h**) Heatmap visualization of the fused feature responses; (**i**) Final fused image.

To further analyze the feature representation capabilities of the proposed dual-branch architecture in fusion tasks, this paper visualizes the intermediate feature responses of the trained model, as shown in Figure 6. In the figure, (a) and (b) represent the infrared image and the visible light image, respectively; (c) and (d) are the grayscale and heatmaps of the feature responses from the Mamba branch; (e) and (f) are the grayscale and heatmaps of the feature responses from the INN branch; (g) and (h) are the grayscale and heatmaps of the fused feature responses; and (i) is the final fused image.

Figure 6c,d show that the Mamba branch exhibits strong responses to human targets, building outlines, and the main structural elements of the scene, indicating that it tends to focus more on global structure and salient information. Figure 6e,f show that the INN branch’s responses are primarily concentrated in local regions such as tree branches, roof edges, windows, and wall boundaries, indicating that it places greater emphasis on texture, edges, and detail representation. As shown in Figure 6g,h, the fused feature responses simultaneously preserve information regarding salient targets, structural elements, and local textures, demonstrating that the two branches provide complementary representations. The final output in Figure 6i further demonstrates that the proposed method can enhance infrared salient objects while preserving visible light background details, thereby validating the rationality and effectiveness of the Mamba-INN dual-branch design.

It should be noted that Figure 5 and Figure 6 are primarily used to qualitatively explain the mechanism of feature division in the two-branch architecture, rather than as independent quantitative evaluation metrics. Quantitative performance is still evaluated collectively using EN (Entropy), SD (Standard Deviation), SF (Spatial Frequency), MI (Mutual Information), SCD (Sum of Correlations of Differences), VIF (Visual Information Fidelity), Qabf, and SSIM (Structural Similarity Index), as described in Section 4.

### 3.4. Decoder

The core function of the decoder is to reconstruct a high-quality fused image from the preliminary fused features $F_{fuse}$ [28]. To achieve a lightweight design for the decoder, this paper abandons the complex multi-stage upsampling, feature enhancement and skip-connection modules found in traditional decoders. Instead, it adopts a single-stage refinement architecture with a bias-free design, which significantly reduces the parameter overhead and computational complexity on the decoding side while ensuring reconstruction quality. The decoder primarily consists of three components: a 1 × 1 convolutional dimension-reduction module, a simplified Transformer block refinement module, and a linear projection output module. The core design philosophy is to achieve an efficient mapping from the high-dimensional fused feature space to the low-dimensional image space by minimizing computational effort.

The decoder employs a bias-free design in all convolutional modules, further reducing the number of model parameters; the single-stage refinement structure ensures fast inference speeds for the decoder, aligning with the goal of a lightweight design for the entire network.

### 3.5. Training Objective

This paper employs an unsupervised training approach that does not require real fused images as supervision labels. The training process is divided into two stages: the first stage involves image self-reconstruction training, and the second stage involves image fusion training. Through these two stages, the model first learns stable feature extraction and reconstruction capabilities, and then further optimizes cross-modal fusion performance.

In the first stage, only the encoder and decoder are trained. Given a visible-light image $I_{vis}$ and an infrared image $I_{ir}$, the encoder extracts both basic and detailed features from the two modalities, and the decoder then reconstructs ${\hat{I}}_{vis}$ and ${\hat{I}}_{ir}$. The loss function for this stage primarily consists of reconstruction loss, gradient loss, and feature decoupling loss:

(8) $$L_{stage1}=L_{rec}+\lambda_{1}L_{grad}+\lambda_{2}L_{decomp}$$

where the reconstruction loss $L_{rec}$ is used to ensure pixel-level and structural consistency between the reconstructed image and the input image, and is composed of both the MSE loss and the SSIM loss:

(9) $$L_{rec}=L_{mse}(I_{vis},{\hat{I}}_{vis})+5L_{ssim}(I_{vis},{\hat{I}}_{vis})+L_{mse}(I_{ir},{\hat{I}}_{ir})+5L_{ssim}(I_{ir},{\hat{I}}_{ir})$$

Gradient loss $L_{grad}$ is used to ensure that the reconstructed image preserves the edge and texture information present in the source image. It is defined as:

(10) $$L_{grad}=\|\nabla I_{vis}-\nabla {\hat{I}}_{vis}{\|}_{1}$$

In addition, to establish a clearer functional division between basic features and detailed features, this paper introduces a feature decoupling loss:

(11) $$L_{decomp}=\frac{\mathrm{cc}{\left(F_{vis}^{D},F_{ir}^{D}\right)}^{2}}{1.01+\mathrm{cc}(F_{vis}^{B},F_{ir}^{B})}$$

where $F_{vis}^{B}$ and $F_{ir}^{B}$ represent the basic features of the visible-light image and the infrared image, respectively; $F_{vis}^{D}$ and $F_{ir}^{D}$ represent the detailed features of the two modalities, respectively; and cc(⋅) represents the correlation metric function. This loss function encourages the basic branch to learn structural information shared by both modalities, while enabling the detail branch to better preserve modality-specific texture and edge information.

In the second stage, the encoder, decoder, and both the base feature fusion layer and the detail feature fusion layer are trained together. The base features and detail features from the two modalities are fed into their respective fusion layers to produce fused base features and fused detail features, which are then used by the decoder to generate the fused image $I_{f}$. The total loss function for this stage is:

(12) $$L_{stage2}=L_{fusion}+\lambda_{2}L_{decomp}$$

where $L_{fusion}$ represents the fusion loss, which is used to ensure that the fused image retains both the salient object information from the infrared image and the textural details from the visible-light image; $L_{decomp}$ is used to maintain the complementary decomposition relationship between the basic features and the detailed features.

Based on the training code settings, the total number of training epochs in this paper is 120, with the first 40 epochs constituting the self-reconstruction training phase and the remaining 80 epochs constituting the fusion training phase. The loss weight is set to $\lambda_{1}=5$, $\lambda_{2}=2$. The model is trained using the Adam optimizer with an initial learning rate of 1 × 10^−4^ and a batch size of 2. The learning rate is halved every 20 epochs, and gradient clipping is enabled to improve training stability.

### 3.6. Evaluation Protocol

To comprehensively evaluate the performance of the proposed method in multimodal image fusion tasks, this paper conducts a quantitative assessment based on information retention, detail representation, structural consistency, visual fidelity, and perceptual quality. Specifically, this paper employs EN, SD, SF, MI, SCD, VIF, Qabf, SSIM, and NIQE as evaluation metrics [29,30,31,32].

EN is used to measure the amount of information contained in the fused image; a higher value indicates that the fused image retains more information. SD reflects the degree of dispersion in the image’s grayscale distribution; a higher value typically indicates stronger image contrast. SF is used to evaluate the degree of spatial variation in the image; a higher value indicates that the image contains more edge and texture details. MI (Mutual Information) is used to measure the amount of information inherited by the fused image from the source images; a higher value indicates that the source image information is more fully preserved. SCD is used to evaluate the ability of the fused image to preserve both the differences and complementary information from the source image; a higher value indicates that the fused image better retains the complementary features of the source image. VIF is used to measure the degree to which visual information is preserved in the fused image; a higher value indicates higher visual information fidelity. Qabf is used to evaluate the ability to transfer edge information from the source image to the fused image; a higher value indicates better preservation of edges and textures. SSIM is used to measure the structural consistency between the fused image and the source image; a higher value indicates more complete preservation of structural information. In addition to the traditional fusion evaluation metrics mentioned above, this paper further introduces NIQE as a perceptual quality evaluation metric. NIQE (Natural Image Quality Evaluator) is a reference-free image quality evaluation metric used to measure the natural statistical characteristics and perceived quality of an image; a smaller value indicates better image quality.

In summary, EN, SD, SF, MI, SCD, VIF, Qabf, and SSIM are all positive metrics, meaning that higher values indicate better fusion performance; NIQE is a negative metric, meaning that lower values indicate better perceived quality.

## 4. Experiments

In this section, the effectiveness of the proposed lightweight Mamba-INN dual-branch fusion network is evaluated on both infrared-visible image fusion and medical image fusion tasks. The experiments are designed from four perspectives: quantitative comparison, qualitative visual evaluation, lightweight performance analysis, and ablation study. Through these evaluations, we examine not only the fusion quality of the proposed method but also its computational efficiency and deployment potential. To ensure fairness, other comparison models, such as CDDFuse, were retrained using the same training set, input resolution, number of training iterations, and hardware environment as our proposed model. All complexity and speed metrics were recalculated for the same input size.

### 4.1. Experimental Set-Up

The model was trained on an NVIDIA RTX 3060 GPU. To comprehensively evaluate the generalization ability of the proposed method, experiments were conducted on both infrared-visible and medical multimodal image fusion datasets. For infrared-visible image fusion, the MSRS [33], TNO [34], and RoadScene [35] datasets were adopted. Among them, MSRS was used for training and testing, while TNO and RoadScene were mainly used to assess the cross-scene generalization performance of the model [1,33,35,36]. For medical image fusion, three representative modality combinations were selected from the Harvard Medical School database, including MRI-CT, MRI-PET, and MRI-SPECT image pairs [7].

### 4.2. Comparative Models

To comprehensively evaluate the fusion performance of the proposed method, this paper selects several classical and state-of-the-art methods for comparison, including DenseFuse [2], DIDFuse [3], U2Fusion [4], SDNet [9], RFNet [6], TarDAL [5], DeFusion [37], ReCoNet [21], CoCoNet [38], and CDDFuse [1].

#### 4.2.1. Quantitative Comparison

In the task of infrared-visible light image fusion, this paper quantitatively evaluates the proposed method on the MSRS, TNO, and RoadScene datasets and compares it with methods such as DIDFuse, U2Fusion, SDNet, TarDAL, DeFusion, ReCoNet, and CDDFuse.

As shown in Table 1, the proposed method achieves competitive performance across all three infrared-visible datasets. On the MSRS dataset, our method obtains strong results in terms of MI, SF, VIF, and Qabf, suggesting that it can effectively integrate salient infrared targets with texture details from visible images. Compared with CDDFuse, the proposed method does not rank first on every single metric, but it maintains comparable fusion quality with a much smaller model size and lower computational cost. This demonstrates that the proposed architecture achieves a favorable balance between fusion performance and model complexity.

On the TNO dataset, our method also demonstrates strong generalization capabilities. This dataset contains a large number of nighttime, low-light, and complex background scenes, which place high demands on target enhancement and detail preservation. Experimental results show that our method maintains stable performance on metrics such as MI, SF, VIF, and Qabf, indicating that the Mamba-INN dual-branch architecture effectively balances global structural modeling and local detail preservation.

**Figure 7:**
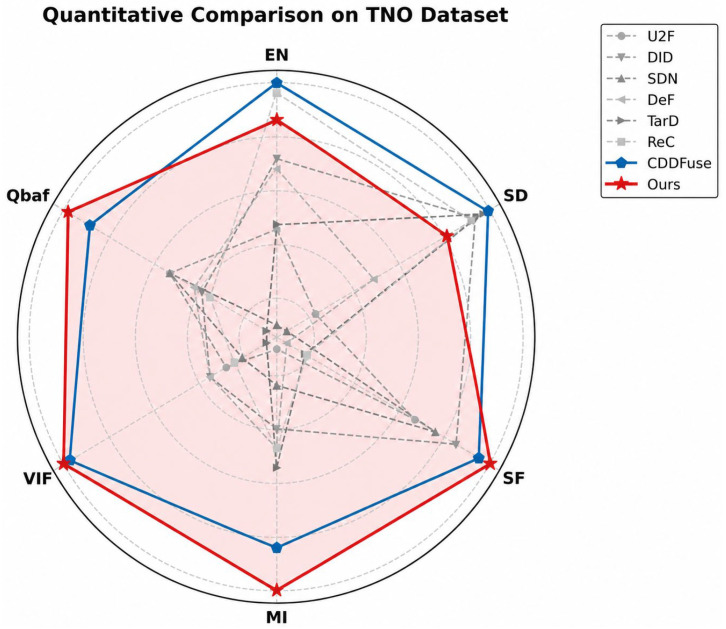
Radar chart comparison of normalized quantitative metrics on the TNO dataset. Each axis denotes one evaluation metric, and a larger enclosed area indicates more balanced overall fusion performance.

To further illustrate the overall performance on the TNO dataset, a radar chart of the main evaluation metrics is presented in Figure 7. Each axis on the radar chart represents a normalized evaluation metric; the larger the area enclosed by the lines, the more balanced the method’s overall performance across multiple metrics. The proposed method covers a relatively large area across multiple metrics and performs particularly well in MI, SF, VIF, and Qabf. This indicates that the method achieves a balanced fusion result in terms of information preservation, detail representation, and edge structure maintenance. Although CDDFuse still has advantages in certain metrics such as EN and SD, the proposed method achieves comparable or better performance on several key perceptual and structural metrics with much lower computational complexity.

On the RoadScene dataset, our method continues to show stable quantitative performance. RoadScene contains complex traffic scenes with roads, vehicles, pedestrians, buildings, and varying illumination conditions. These characteristics require the fusion model to preserve background structures while enhancing salient infrared targets. The results show that the proposed method maintains a good level of information content and image clarity, while avoiding obvious structural distortion. Compared with traditional or lightweight methods, the proposed method achieves stronger performance in information retention and detail representation.

A comprehensive analysis of results across three IR-visible light datasets reveals that our method does not achieve the best performance on every individual metric, but rather strikes a more balanced outcome between fusion performance and model complexity. Compared to CDDFuse, our method significantly reduces the number of parameters and FLOPs by simplifying the Mamba global branch, streamlining the INN detail branch, and compressing the decoding structure, while maintaining competitive fusion quality. Therefore, our method is more suitable for IR-Vis fusion scenarios with resource constraints or high real-time requirements.

To further evaluate cross-modal adaptability, experiments were conducted on MRI-CT, MRI-PET, and MRI-SPECT fusion tasks. Medical image fusion requires preserving anatomical structures and tissue boundaries while integrating functional information. MRI provides soft-tissue details, CT highlights bone structures, and PET/SPECT reflects metabolic or perfusion responses. Thus, effective fusion should balance structural detail preservation with functional information integration.

To distinguish cross-task generalization from task-specific adaptation, two experimental settings were adopted. “Ours” denotes the model trained on the infrared-visible fusion task and directly tested on medical image fusion datasets, which evaluates the transferability of the proposed architecture. “Ours*” denotes the model retrained on medical image datasets, which evaluates its adaptability to medical modality distributions. Similarly, CDDFuse* represents the retrained version of CDDFuse on medical image fusion datasets. This setting provides a clearer comparison between inherent architectural generalization and task-specific optimization.

As shown in Table 2, the proposed method achieves stable quantitative results across MRI-CT, MRI-PET, and MRI-SPECT fusion tasks. For MRI-CT fusion, our method effectively combines soft-tissue information from MRI with high-density structural information from CT, producing fused images with clear contours and comprehensive anatomical representation. For MRI-PET and MRI-SPECT fusion, the model introduces significant functional responses from PET or SPECT while preserving the structural details of MRI. These results suggest that the proposed Mamba-INN dual-branch architecture can balance structural preservation and functional information integration.

The comparison between Ours and Ours* further shows that retraining on medical datasets can improve the model’s adaptation to medical modality distributions. Nevertheless, even without medical-specific retraining, the directly transferred model still achieves competitive performance, which indicates that the proposed dual-branch modeling strategy has a certain degree of cross-modal transferability. The Mamba-inspired branch contributes to large-scale structural dependency modeling, while the lightweight INN branch helps retain local edges and fine details. Their complementary roles allow the model to adapt to different types of multimodal image fusion tasks.

To further validate the generalization ability of the proposed model on unseen datasets, we conducted a zero-shot evaluation on the official LLVIP [39] test set. No additional training or fine-tuning was performed on the model during the evaluation. The results are shown in Table 3. As can be seen from the table, although our model was not trained on the LLVIP dataset, its fusion performance still demonstrates good stability. For the EN metric, Ours achieved a score of 7.35, only slightly lower than CDDFuse’s 7.44; while it is slightly lower than CDDFuse on the SD metric, it achieves 0.68 and 0.91 on structural and edge preservation metrics such as Qabf and SSIM, respectively, demonstrating an advantage in preserving detail and structural information. This indicates that the proposed Mamba-INN dual-branch architecture can maintain relatively stable fusion quality when faced with unseen infrared-visible light datasets, demonstrating a certain degree of cross-dataset generalization capability.

To further evaluate the naturalness of the fused images, we employed the NIQE metric for testing. Table 4 lists the NIQE test results for different methods across four datasets, comparing CDDFuse with our proposed method (Ours). The experimental results show that our method achieves NIQE values comparable to those of CDDFuse on most datasets, indicating that it delivers stable and competitive performance in preserving image naturalness.

In summary, the proposed method demonstrates good stability and adaptability in medical image fusion. Although it does not obtain the best result on every individual metric, it maintains competitive fusion quality with a significantly smaller number of parameters and lower computational cost than CDDFuse and CDDFuse*. These results indicate that the proposed lightweight architecture is not only effective for infrared-visible fusion but also has potential for medical multimodal fusion scenarios where computational efficiency and inference speed are important.

#### 4.2.2. Qualitative Comparison

To provide a more intuitive evaluation of visual fusion quality, qualitative comparisons were conducted for both infrared-visible and medical image fusion tasks. Compared with quantitative metrics, visual comparison can better reflect target saliency, texture preservation, structural clarity, contrast balance, and artifact suppression in fused images.

**Figure 8:**
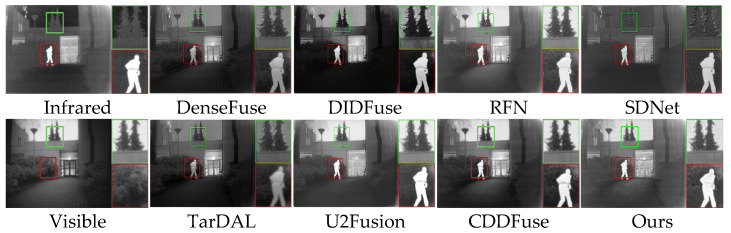
Visual comparison between different methods on TNO dataset.

For the infrared-visible fusion task on the TNO dataset, the visual results are shown in Figure 8. The columns in the figure correspond to the source image and the results of different fusion methods, respectively, and are used to compare the saliency of infrared targets and the ability to preserve visible-light textures. Some images in the comparison were obtained from CoCoNet [38]. It can be observed that different methods show different fusion tendencies. Some methods enhance infrared targets effectively but weaken visible background textures and edge structures. Other methods preserve part of the visible details but fail to sufficiently highlight salient infrared targets, resulting in weak contrast between targets and background. In contrast, the proposed method enhances infrared targets while retaining texture and structural information from visible images. In low-light or complex-background regions, road surfaces, building boundaries, and object contours are clearly preserved, and no obvious brightness imbalance or over-smoothing can be observed.

A closer inspection of the locally enlarged regions further confirms the advantage of the proposed method. Traditional fusion methods tend to produce blurred edges, discontinuous textures, or insufficient local contrast in detail-rich regions. CDDFuse achieves strong visual quality, but its network structure is relatively complex. The proposed method obtains comparable visual results with a much more compact architecture. This benefit mainly comes from the cooperative design of the lightweight Mamba-inspired branch and the INN detail branch. The Mamba-inspired branch helps maintain large-scale structural consistency, while the INN branch reduces the loss of high-frequency textures and edges during fusion.

**Figure 9:**
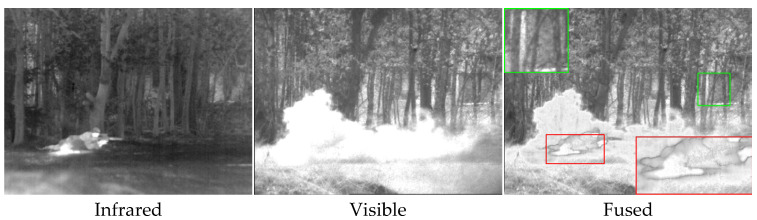

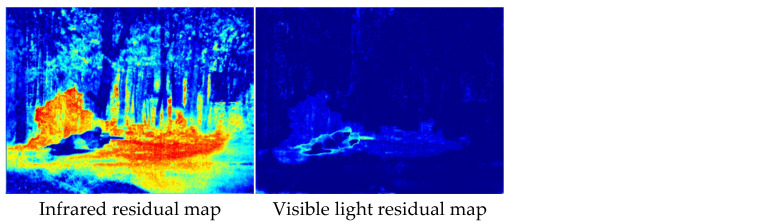
Results of feature fusion and residual analysis for representative samples in the TNO dataset.

To further analyze how information from the two source modalities is inherited in the fused image, residual maps between the fused image and the infrared and visible images are presented in Figure 9. The top row shows the infrared image, the visible light image, and the fused image; the bottom row shows the residual maps between the fused image and the infrared image, and between the fused image and the visible light image, respectively. The residual map between the fused image and the infrared image mainly highlights background structures and texture regions, indicating that visible-light details are effectively introduced into the fused result. Meanwhile, the residual map between the fused image and the visible image is mainly concentrated around foreground target contours and their surrounding regions, suggesting that infrared thermal targets are successfully enhanced. These residual distributions provide additional evidence that the proposed method can achieve complementary fusion between infrared saliency and visible texture information.

**Figure 10:**
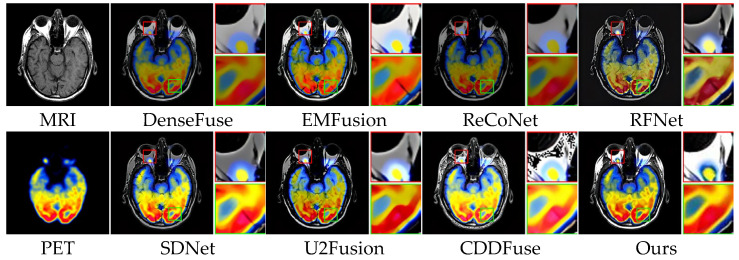
Visual comparison for “MRI-PET-16” in MRI-PET MIF.

For medical image fusion, the visual comparisons are shown in Figure 10. MRI images primarily show anatomical structures and tissue boundaries, while PET images primarily provide information on functional metabolism. Different methods present obvious differences in anatomical structure preservation and functional information integration. Some methods enhance high-response regions from functional modalities but tend to weaken MRI structural details or blur tissue boundaries. Other methods preserve MRI structures but fail to adequately represent PET or SPECT functional responses. In contrast, the proposed method preserves anatomical structures and tissue boundaries from MRI while incorporating complementary information from CT, PET, or SPECT. The resulting fused images exhibit clearer structural contours and more complete functional representation.

For MRI-CT fusion, the proposed method simultaneously retains MRI soft-tissue structures and CT high-density bone boundaries, improving the structural clarity and stability of the fused images. For MRI-PET and MRI-SPECT fusion, the proposed method highlights functional response regions while preserving MRI anatomical details, avoiding excessive smoothing of functional information or masking of structural information. These visual results indicate that the proposed method is suitable not only for target-texture fusion in infrared-visible scenarios but also for structure-function fusion in medical multimodal imaging.

Overall, the qualitative results show that the proposed method achieves stable and balanced visual performance. Compared with traditional or lightweight fusion methods, it better preserves edges, textures, and structural information. Compared with more complex models such as CDDFuse, it achieves comparable visual quality with significantly fewer parameters and lower computational complexity. This further confirms the effectiveness of the proposed lightweight Mamba-INN dual-branch architecture.

### 4.3. Lightweight Performance Comparison

To evaluate the lightweight advantage of the proposed model, the number of parameters and FLOPs were compared with those of mainstream image fusion methods. The results are reported in Table 5. The parameter and FLOP values of some comparison methods were obtained from CoCoNet [38].

As shown in Table 5, the proposed model contains only 0.24 M parameters and requires 24.04 GFLOPs. Both values are substantially lower than those of U2Fusion, DenseFuse, SwinFusion, FMamba-S, FMamba-L, CDDFuse, and other comparison methods. Compared with CDDFuse, a representative state-of-the-art dual-branch fusion model, our method reduces the parameter count by approximately 79.8% and the computational complexity by approximately 79.5%. This means that the proposed model achieves an overall complexity reduction of nearly 80% while maintaining competitive fusion quality.

**Figure 11:**
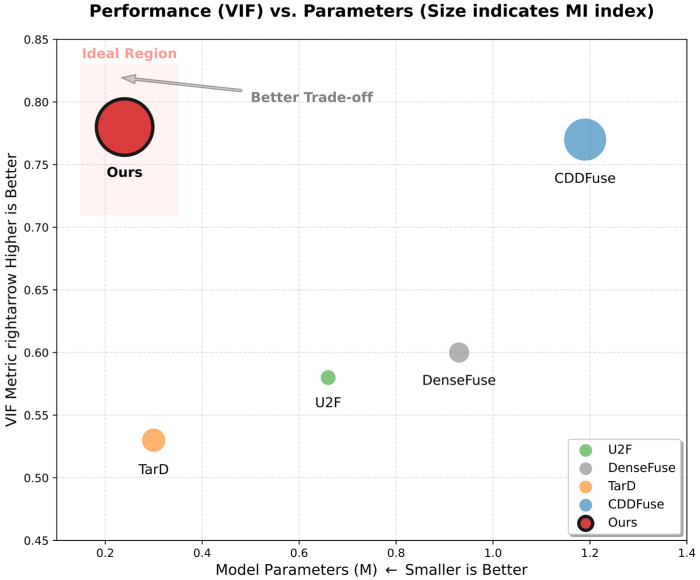
Comprehensive comparison of different methods in terms of parameter quantity, VIF and MI indicators.

To further analyze the relationship between model complexity and fusion performance, Figure 11 provides a comprehensive comparison of the number of parameters, VIF, and MI metrics across different methods. The horizontal axis represents the number of model parameters; the further to the left, the lighter the model. The vertical axis represents VIF; higher values indicate better visual information fidelity. The size of the bubbles represents MI; larger bubbles indicate that more mutual information has been preserved. Our method achieves high VIF values and large MI bubbles even with a low number of parameters, indicating that it maintains good information retention and fusion quality while significantly reducing model complexity.

It is worth noting that, compared with CDDFuse, the proposed method achieves a comparable or higher VIF value with far fewer parameters while maintaining a high MI level. This demonstrates that the proposed lightweight dual-branch framework can effectively reduce model scale without significantly compromising fusion quality. The performance-complexity comparison confirms that the proposed method has strong practical potential for deployment on computationally constrained platforms.

The lightweight advantage mainly comes from three aspects. First, the simplified Mamba-inspired branch reduces the computational burden of global modeling. Second, the streamlined INN branch preserves local details with limited parameter overhead. Third, the compact encoder–decoder structure, module reuse strategy, and low-dimensional channel design jointly reduce redundant computation in the feature extraction, fusion, and reconstruction stages. These designs allow the model to achieve both low complexity and high fusion performance, making it suitable for real-time image fusion on embedded devices, mobile terminals, and other resource-limited platforms.

### 4.4. Computational Efficiency and Scalability

To further assess the computational efficiency of the proposed model, this section analyzes its parameter count, FLOPs, inference latency, and frame rate under different input resolutions. The comparison in Table 5 has already shown that the proposed method has significantly fewer parameters and lower computational complexity than most mainstream fusion methods. In particular, compared with CDDFuse, the proposed method reduces both parameters and FLOPs by about 80%, which verifies the effectiveness of the simplified Mamba branch, lightweight INN branch, and module reuse strategy.

In addition to fixed-resolution complexity comparison, cross-resolution efficiency tests were conducted to evaluate the scalability of the proposed model. Four input sizes were used: 128 × 128, 256 × 256, 512 × 512, and 1024 × 1024. The parameter count, FLOPs, inference latency, and FPS were recorded for each resolution. All tests were conducted on an NVIDIA GeForce RTX 3060 Laptop GPU with a batch size of 1. To obtain stable latency measurements, each input size was tested after 30 warm-up runs, followed by 100 formal inference runs. The final results were averaged. FLOPs were measured using the THOP tool, with two single-channel images as model input.

As shown in Table 6, the parameter count remains constant at 0.24 M as the input resolution increases. This demonstrates that the model size is independent of spatial resolution and confirms the structural compactness of the proposed network. FLOPs increase steadily with input size. When the input resolution increases from 128 × 128 to 256 × 256, the number of pixels increases by four times, and the FLOPs rise from 6.01 G to 24.04 G. When the resolution further increases to 512 × 512 and 1024 × 1024, the FLOPs reach 96.16 G and 384.63 G, respectively. This trend is consistent with the increase in spatial resolution, indicating that the computational growth of the proposed model is stable and predictable.

In terms of inference speed, the proposed method achieves an average latency of 23.00 ms at 128 × 128 resolution, corresponding to 43.48 FPS. This indicates that the model can meet high real-time requirements under small input sizes. At the commonly used 256 × 256 resolution, the latency is 94.74 ms, and the frame rate reaches 10.56 FPS, which still reflects reasonable inference efficiency on a laptop-class GPU. When the input size increases to 512 × 512 and 1024 × 1024, the latency rises to 381.35 ms and 1540.04 ms, while the FPS decreases to 2.62 and 0.65, respectively. Although high-resolution inputs still introduce considerable computational pressure, the model does not show abnormal complexity growth.

To further address deployment requirements in resource-constrained scenarios, this paper builds upon existing resolution scalability experiments by adding CPU-only constrained inference tests. Unlike the multi-resolution inference experiments in Table 6, which were conducted using an NVIDIA GeForce RTX 3060 Laptop GPU, this experiment is performed entirely in a CPU environment without GPU acceleration to more closely simulate inference conditions under computational resource constraints. During testing, the input resolution was fixed at 256 × 256, the batch size was set to 1, and the number of CPU threads was set to 1, 2, 4, and 8, respectively, to analyze the model’s inference latency, frame rate, model storage size, and additional memory usage under different computational resource configurations. It should be noted that this experiment is not equivalent to deployment testing on real embedded hardware platforms; its purpose is to provide a reproducible analysis of CPU-constrained inference, thereby further validating the deployment potential of the proposed lightweight model.

Table 7 presents the results of the CPU-only constrained inference tests. As shown, the storage size of the proposed model is only 0.9925 MB, indicating that the model has a small storage footprint and is suitable for model loading and deployment in resource-constrained scenarios. Under single-threaded CPU conditions, the model’s average inference latency was 2605.30 ms, with a frame rate of 0.3838 FPS; when the number of CPU threads was increased to 2, the inference latency decreased to 1575.28 ms, and the frame rate improved to 0.6348 FPS; when the number of threads was further increased to 4, the inference latency decreased to 862.23 ms, and the frame rate increased to 1.1598 FPS; under 8-thread conditions, the model achieved the best inference efficiency under the experimental setup, with the average inference latency further reduced to 497.23 ms and the frame rate increased to 2.0111 FPS. Compared to the single-threaded configuration, inference latency under the 8-thread condition was reduced by approximately 80.9%, indicating that the model can effectively leverage multi-threaded CPU computing resources.

Furthermore, in terms of additional memory usage, the memory overhead under different thread configurations remained within a manageable range, at 345.30 MB, 247.92 MB, 266.86 MB, and 372.38 MB, respectively. Although the inference speed under CPU-only conditions remains lower than that on GPU platforms, these experimental results demonstrate that the method proposed in this paper possesses stable CPU inference capabilities and good multithreading scalability while maintaining a compact model size. Therefore, combined with the analysis in Table 5 and Table 6 regarding the number of parameters, FLOPs, and inference efficiency at different resolutions, this further demonstrates that the lightweight Mamba-INN dual-branch network proposed in this paper has certain application potential in resource-constrained deployment scenarios.

Overall, the proposed method exhibits good real-time potential for small- and medium-resolution inputs and maintains stable computational scalability at higher resolutions. It should be noted that inference latency depends on multiple factors, including hardware platform, deep learning framework, operator optimization, and implementation details. Therefore, the latency results reported here are mainly used to analyze the computational trend of the model under different input scales rather than to provide hardware-independent speed conclusions. Nevertheless, the results confirm that the proposed Mamba-INN dual-branch framework provides a compact and efficient model basis for real-time or near-real-time multimodal image fusion.

**Figure 12:**
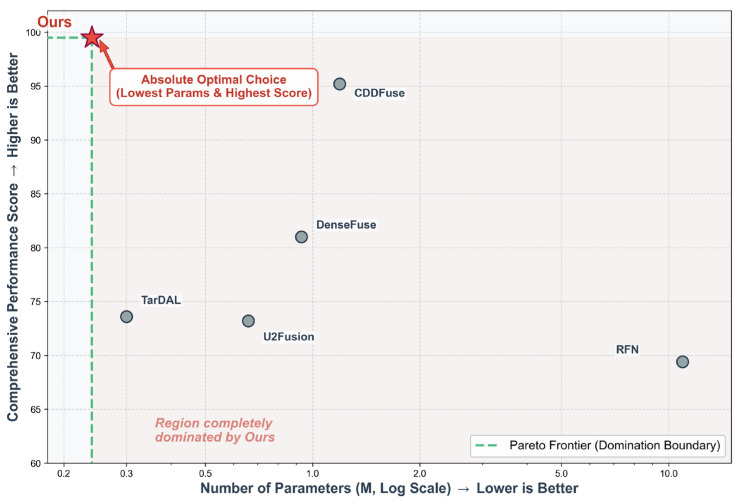
Pareto front analysis of efficiency and performance on an MRI-PET dataset. The composite score is calculated based on six normalized objective metrics. The green dashed line indicates the Pareto frontier, highlighting that our method achieves optimal fusion performance with the fewest parameters. The red star represents our proposedmethod, which achieves the highest comprehensive performance score with the lowest number of parameters, making it the absolute optimal choice among all compared methods.

Furthermore, a Pareto front analysis was conducted on the MRI-PET dataset to evaluate the relationship between model complexity and fusion performance, as shown in Figure 12. The horizontal axis represents the number of model parameters and is plotted on a logarithmic scale; the vertical axis represents the overall performance score, which is calculated based on multiple normalized fusion metrics. The green dashed line indicates the Pareto front; methods located on this front achieve an optimal trade-off between the number of parameters and performance. Our method lies on the Pareto front and achieves a high overall score with a small number of parameters, demonstrating its advantages in terms of model efficiency and performance stability for medical image fusion tasks.

Since the magnitude and distribution ranges of the aforementioned six metrics vary greatly (e.g., SF is typically greater than 20, while Qbaf is less than 1), simply summing them lacks scientific justification. Therefore, we propose a method for calculating a normalized Comprehensive Performance Score. First, we use Min-Max Normalization to map the *i*-th metric $m_{i,j}$ of the *j*-th method to the interval [0, 1]:

(13) $${\hat{m}}_{i,j}=\frac{m_{i,j}-min(m_{i})}{max(m_{i})-min(m_{i})}$$

where $m_{i,j}$ represents the raw score of the *i*-th method on the *j*-th evaluation metric, ${\hat{m}}_{i,j}$ represents the normalized score, and m represents the number of evaluation metrics. $S_{i}$ represents the overall evaluation score for method *i*. For metrics where higher values are better, positive normalization is applied.

Next, we calculate the average of the normalized metrics and map them linearly to a standard 100-point scale ranging from 60 to 100 to enhance the clarity of the visualization. The final composite $S_{j}$ for the *j*-th method is calculated as follows (where *N* = 6 is the total number of selected metrics):

(14) $$S_{j}=60+40\times \left(\frac{1}{N}\sum_{i=1}^{N}{\hat{m}}_{i,j}\right)$$

As illustrated in Figure 12, the horizontal axis represents the number of parameters on a logarithmic scale, while the vertical axis denotes the comprehensive performance score. The proposed method lies on the Pareto frontier and forms a favorable boundary in terms of both efficiency and performance. Specifically, it achieves the highest composite score of 99.5 while using only 0.24 M parameters, making it the lightest model among the compared methods. In contrast, CDDFuse obtains the second-highest score of 95.2 but requires 1.19 M parameters, nearly five times that of the proposed model. This result further confirms that the proposed decoupled Mamba-INN architecture can achieve strong fusion performance with a substantially reduced model size.

To provide a clearer numerical comparison corresponding to the Pareto analysis, Table 8 reports the parameter counts and comprehensive performance scores of different methods on the MRI-PET dataset. The comprehensive score is calculated based on six normalized evaluation metrics, including SF, MI, SCD, VIF, Qabf, and SSIM. This table allows a direct comparison between fusion performance and model complexity, thereby further illustrating the efficiency advantage of the proposed method.

### 4.5. Ablation Studies

To validate the effectiveness of each key module, we conducted ablation experiments on the infrared-visible light image fusion task, with the results shown in Table 8. In this table, “heavy baseline” refers to the baseline model without any lightweight design; “w/o Mamba,” “w/o INN,” and “w/o module reuse” denote the removal of the Mamba branch, the INN branch, and the module reuse strategy, respectively; and “Full module” refers to the complete model proposed in this paper.

As shown in Table 9, the heavy baseline has the highest model complexity, with 1.19 M parameters and 116.85 G FLOPs, but its fusion performance is not optimal. In contrast, the Full module contains only 0.24 M parameters and 24.04 G FLOPs, representing reductions of approximately 79.83% and 79.43% compared to the heavy baseline, respectively. It achieves the best results on Qabf while maintaining competitive performance on MI and VIF, indicating that our method can maintain stable fusion quality while significantly reducing complexity.

Specifically, the w/o Mamba variant achieves the highest MI value, but its Qabf is lower than that of the Full module, indicating that the model’s ability to model global structure and maintain edge consistency declines without the Mamba branch. The w/o INN variant achieves the highest SF, but its Qabf remains lower than that of the Full module, suggesting that higher spatial frequency does not necessarily correspond to better edge information propagation, and that the INN branch plays a positive role in preserving details and textures. For the w/o module reuse variant, both the number of parameters and FLOPs are higher than those of the Full module, and the VIF drops to 0.76, indicating that the module reuse strategy can effectively reduce redundant computations while maintaining consistency in the feature extraction and fusion processes.

In summary, the Mamba branch, INN branch, and module reuse strategy play crucial roles in global structure modeling, local detail preservation, and model lightweighting, respectively. The full model achieved optimal Qabf and stable overall performance with the lowest number of parameters and computational cost, validating the effectiveness of the proposed lightweight Mamba-INN dual-branch architecture.

## 5. Conclusions

To address the critical challenge of balancing performance and computational efficiency in lightweight image fusion tasks, this paper proposes a lightweight dual-branch feature fusion network based on simplified Mamba blocks and lightweight reversible modules. Centered on lightweight design, this network reconfigures the encoding and decoding framework and introduces a decoupled dual-branch feature extraction architecture. The base branch efficiently captures global structural information using simplified Mamba blocks, while the detail branch relies on a lightweight INN to achieve lossless extraction of high-frequency details. By combining a module reuse strategy to perform feature fusion and image reconstruction, the network ensures the effectiveness of feature extraction and fusion while strictly controlling the number of model parameters and computational complexity. Experimental results on the TNO and RoadScene infrared-visible light fusion datasets, as well as the MRI-CT, MRI-PET, and MRI-SPECT multimodal medical image fusion datasets, demonstrate that the proposed method outperforms mainstream methods such as U2F and CDDFuse in core metrics including information retention and detail representation. Furthermore, the model has only 0.24 million parameters and computational cost of 24.04 GFLOPs, achieving an approximately 80% reduction in complexity compared to CDDFuse. Ablation experiments further validated the rationality and effectiveness of each core module and their combinations. The method proposed in this paper successfully achieves a balance between performance and computational efficiency in lightweight image fusion, providing an effective solution for multimodal image fusion in real-time processing scenarios such as edge devices and mobile terminals. Future research could focus on optimizing multi-scale feature interaction mechanisms, expanding multimodal fusion scenarios, and model quantization and deployment to enhance the model’s representational capabilities and engineering practicality.

## Figures and Tables

**Table 1:** Comparison of quantitative infrared-visible fusion (IVF) results. Bold font indicates the best performance; underlined font indicates the second-best performance.

**Dataset: MSRS Infrared-Visible Fusion Dataset**								
	EN	SD	SF	MI	SCD	VIF	Qbaf	SSIM
U2F	5.37	25.52	9.07	1.40	1.24	0.54	0.42	0.77
DID	4.27	31.49	10.15	1.61	1.11	0.31	0.20	0.24
SDN	5.25	17.35	8.67	1.19	0.99	0.50	0.38	0.72
DeF	6.46	37.63	8.60	2.16	1.35	0.77	0.54	0.94
TarD	5.28	25.22	5.98	1.49	0.71	0.42	0.18	0.47
ReC	6.61	43.24	9.77	2.16	1.44	0.71	0.50	0.85
CDDFuse	**6.70**	**47.81**	12.03	**3.46**	**1.68**	**1.01**	0.67	0.99
Ours	6.69	46.77	**12.09**	3.43	1.64	0.99	**0.70**	**0.99**
**Dataset: TNO Infrared-Visible Fusion Dataset**								
	EN	SD	SF	MI	SCD	VIF	Qbaf	SSIM
U2F	6.83	34.55	11.52	1.37	1.71	0.58	0.44	0.99
DID	6.97	45.12	12.59	1.70	1.71	0.60	0.40	0.81
SDN	6.64	32.66	12.05	1.52	1.49	0.56	0.44	1.00
DeF	6.95	38.41	8.21	1.78	1.64	0.60	0.41	0.96
TarD	6.84	45.63	8.68	1.86	1.52	0.53	0.32	0.88
ReC	7.10	44.85	8.73	1.78	1.70	0.57	0.39	0.88
CDDFuse	**7.12**	**46.00**	13.15	2.19	**1.76**	0.77	0.54	**1.03**
Ours	7.03	42.55	**13.44**	**2.37**	1.63	**0.78**	**0.57**	1.00
**Dataset: RoadScene Infrared-Visible Fusion Dataset**								
	EN	SD	SF	MI	SCD	VIF	Qbaf	SSIM
U2F	7.09	38.12	13.25	1.87	1.70	0.60	0.51	0.97
DID	7.43	51.58	14.66	2.11	1.70	0.58	0.48	0.86
SDN	7.14	40.20	13.70	2.21	1.49	0.60	0.51	**0.99**
DeF	7.23	44.44	10.22	2.25	1.69	0.63	0.48	0.89
TarD	7.17	47.44	10.83	2.14	1.55	0.54	0.40	0.88
ReC	7.36	52.54	10.78	2.18	1.74	0.59	0.43	0.88
CDDFuse	**7.43**	**54.66**	**16.36**	2.30	**1.81**	0.69	0.52	0.98
Ours	7.30	48.77	15.61	**2.47**	1.68	**0.70**	**0.57**	0.95

**Table 2:** Comparison of Quantitative Results for medical image fusion (MIF). Bold font indicates the best performance; underlined font indicates the second-best performance. CDDFuse* and Ours* represent the results after training on MIF datasets.

**Dataset: MRI-CT Medical Image Fusion**								
	EN	SD	SF	MI	SCD	VIF	Qbaf	SSIM
TarD	4.75	61.14	28.38	1.94	0.81	0.32	0.35	0.99
RFN	**5.30**	52.95	33.42	1.98	0.58	0.33	0.52	0.49
ReC	4.41	66.96	20.16	2.03	1.24	0.40	0.42	1.29
DeF	4.63	66.38	21.56	2.20	1.12	0.47	0.44	1.29
CDDFuse	4.73	**88.38**	33.82	2.23	**1.74**	0.50	0.59	**1.33**
Ours	4.85	85.92	**37.31**	**2.32**	1.64	**0.53**	**0.64**	1.27
U2F	4.88	52.98	22.54	2.08	0.75	0.37	0.46	0.49
SDN	**5.02**	60.07	29.41	2.14	0.97	0.38	0.47	0.51
EMF	4.76	72.76	22.56	2.34	1.32	0.56	0.49	1.31
CDDFuse*	4.77	**78.99**	38.14	**2.60**	**1.40**	**0.61**	**0.68**	**1.35**
Ours*	4.78	73.40	**39.85**	2.34	0.96	0.48	0.64	1.24
**Dataset: MRI-PET Medical Image Fusion**								
	EN	SD	SF	MI	SCD	VIF	Qbaf	SSIM
TarD	3.81	57.65	23.65	1.36	1.46	0.57	0.58	0.68
RFN	**4.77**	50.57	**29.11**	1.53	0.96	0.39	0.52	0.42
ReC	3.66	65.25	21.72	1.51	1.49	0.44	0.51	1.40
DeF	4.17	64.65	22.35	1.74	1.48	0.58	0.56	1.45
CDDFuse	4.15	**81.49**	28.04	1.86	1.81	0.66	0.65	**1.48**
Ours	4.33	78.84	29.00	**1.98**	**1.81**	**0.70**	**0.70**	1.42
U2F	3.73	57.07	23.27	1.69	1.27	0.40	0.49	1.39
SDN	3.83	61.40	**31.97**	1.71	1.40	0.47	0.57	1.46
EMF	4.21	56.80	26.01	1.82	1.31	0.62	0.67	1.47
CDDFuse*	4.14	**70.55**	29.57	**2.02**	**1.68**	**0.71**	**0.71**	1.51
Ours*	**4.28**	62.62	26.71	1.99	1.39	0.62	0.69	**1.51**
**Dataset: MRI-SPECT Medical Image Fusion**								
	EN	SD	SF	MI	SCD	VIF	Qbaf	SSIM
TarD	3.66	53.46	18.50	1.41	0.90	0.64	0.52	0.36
RFN	**4.39**	44.01	**23.77**	1.60	0.72	0.45	0.58	0.37
ReC	3.22	60.07	17.40	1.50	1.47	0.46	0.54	1.40
DeF	3.81	56.65	15.45	1.80	1.27	0.61	0.56	1.46
CDDFuse	3.82	**71.62**	20.66	1.89	**1.87**	0.65	0.68	**1.47**
Ours	4.04	66.84	21.15	**2.00**	1.83	**0.72**	**0.74**	1.43
U2F	3.47	52.97	19.58	1.68	1.28	0.48	0.57	1.41
SDN	3.43	49.62	**22.20**	1.69	1.09	0.55	0.66	1.48
EMF	3.74	51.93	17.14	1.88	1.12	0.71	0.74	1.49
CDDFuse*	3.82	**58.13**	20.87	**2.47**	**1.34**	**0.97**	**0.78**	**1.49**
Ours*	**3.91**	47.78	17.12	2.02	0.47	0.67	0.72	1.41

**Table 3:** Quantitative results of zero-shot infrared-visible image fusion evaluation on the official LLVIP test set.

Dataset	Method	EN	SD	SF	MI	SCD	VIF	Qabf	SSIM
LLVIP	CDDFuse	7.44	53.21	17.12	2.97	1.42	0.85	0.62	0.89
	Ours	7.35	49.82	17.10	2.58	1.42	0.81	0.68	0.91

**Table 4:** Comparison of NIQE metric (mean ± standard deviation) between CDDFuse and the proposed method on different datasets.

Dataset	Method	NIQE (Mean ± Standard Deviation)
MSRS	CDDFuse	2.8630 ± 0.2546
	Ours	2.9110 ± 0.2892
TNO	CDDFuse	4.2500 ± 0.7339
	Ours	3.7038 ± 0.9423
MRI_PET	CDDFuse	6.6552 ± 0.8956
	Ours	6.8170 ± 0.7912
MRI_SPET	CDDFuse	6.0426 ± 0.8599
	Ours	6.0586 ± 1.0559

**Table 5:** Comparison of lightweight performance results. Bold font indicates the best performance; underlined font indicates the second-best performance.

Method	Params (M)	Flops (G)
U2F	0.66	366.34
DenseFuse	0.93	497.96
GANMcC	1.86	1002.56
RFN	10.94	676.06
SwinFusion	0.97	471.04
TarD	0.30	82.37
CoCoNet	9.13	115.37
FMamba-S	0.61	45.90
FMamba-L	0.65	51.18
CDDFuse	1.19	116.85
Ours	**0.24**	**24.04**

**Table 6:** Test results on the computational efficiency of the method described in this paper under different input resolutions.

Input Size	Params (M)	Flops (G)	Latency (ms)	FPS
128 × 128	0.24	6.01	23.00	43.48
256 × 256	0.24	24.04	94.74	10.56
512 × 512	0.24	96.16	381.35	2.62
1024 × 1024	0.24	384.63	1540.04	0.65

**Table 7:** CPU-only constrained inference evaluation of the proposed method.

CPU Threads	Model Size (MB)	Latency (ms)	FPS	Extra Memory (MB)
1	0.9925	2605.30	0.38	43.48
2	0.9925	1575.28	0.63	10.56
4	0.9925	862.23	1.16	2.62
8	0.9925	497.23	2.01	0.65

**Table 8:** Evaluation of model parameters and composite scores on an MRI-PET dataset. The composite score is calculated based on the normalized results of six key visual and structural metrics. Bold font indicates the best performance; underlined font indicates the second-best performance.

Method	Params (M)	Score
U2F	0.66	73.2
DenseFuse	0.93	81.0
RFN	10.94	69.4
TarD	0.30	73.6
CDDFuse	1.19	95.2
Ours	**0.24**	**99.5**

**Table 9:** Ablation experiments on the IVF mission. Bold font indicates the best performance; underlined font indicates the second-best performance.

	Configuration	SF	MI	VIF	Qbaf	Params (M)	Flops (G)
I	heavy baseline	13.15	2.19	0.77	0.54	1.19	116.85
II	w/o Mamba	13.74	**2.40**	0.78	0.55	0.89	88.31
III	w/o INN	**13.93**	2.35	0.78	0.56	0.53	53.45
IV	w/o module reuse	13.47	2.36	0.76	0.56	0.31	31.62
Full module		13.44	2.37	**0.78**	**0.57**	**0.24**	**24.04**

## Data Availability

The datasets used in this study are all publicly available. This manuscript encompasses all data that were produced or examined throughout the course of this study. Accompanying scripts and computational methods integral to the data’s creation will be made available in due course.

## Abbreviations

The following abbreviations are used in this manuscript:

IVF	Infrared-visible image fusion
MIF	Medical image fusion
IR	Infrared image
VIS	Visible image
MRI	Magnetic resonance imaging
CT	Computed tomography
PET	Positron emission tomography
INN	Invertible neural network
SSM	State space model
FLOPs	Floating-point operations
FPS	Frames per second
EN	Entropy
SD	Standard deviation
SF	Spatial frequency
MI	Mutual information
SCD	Sum of correlations of differences
VIF	Visual information fidelity
SSIM	Structural similarity index measure
DWConv	Depthwise convolution
PWConv	Pointwise convolution
